# Enhancing brain tumor classification in MRI scans with a multi-layer customized convolutional neural network approach

**DOI:** 10.3389/fncom.2024.1418546

**Published:** 2024-06-12

**Authors:** Eid Albalawi, Arastu Thakur, D. Ramya Dorai, Surbhi Bhatia Khan, T. R. Mahesh, Ahlam Almusharraf, Khursheed Aurangzeb, Muhammad Shahid Anwar

**Affiliations:** ^1^Department of Computer Science, King Faisal University, Al-Ahsa, Saudi Arabia; ^2^Department of Computer Science and Engineering, Faculty of Engineering and Technology, JAIN (Deemed-to-be University), Bangalore, India; ^3^Department of Information Science and Engineering, Faculty of Engineering and Technology, JAIN (Deemed-to-be University), Bangalore, India; ^4^School of Science, Engineering and Environment, University of Salford, Manchester, United Kingdom; ^5^Department of Electrical and Computer Engineering, Lebanese American University, Byblos, Lebanon; ^6^Department of Management, College of Business Administration, Princess Nourah Bint Abdulrahman University, Riyadh, Saudi Arabia; ^7^Department of Computer Engineering, College of Computer and Information Sciences, King Saud University, Riyadh, Saudi Arabia; ^8^Department of AI and Software, Gachon University, Seongnam-si, Republic of Korea

**Keywords:** diagnosis of brain tumors, convolutional neural networks, deep learning, classification of medical images, MRI imaging

## Abstract

**Background:**

The necessity of prompt and accurate brain tumor diagnosis is unquestionable for optimizing treatment strategies and patient prognoses. Traditional reliance on Magnetic Resonance Imaging (MRI) analysis, contingent upon expert interpretation, grapples with challenges such as time-intensive processes and susceptibility to human error.

**Objective:**

This research presents a novel Convolutional Neural Network (CNN) architecture designed to enhance the accuracy and efficiency of brain tumor detection in MRI scans.

**Methods:**

The dataset used in the study comprises 7,023 brain MRI images from figshare, SARTAJ, and Br35H, categorized into glioma, meningioma, no tumor, and pituitary classes, with a CNN-based multi-task classification model employed for tumor detection, classification, and location identification. Our methodology focused on multi-task classification using a single CNN model for various brain MRI classification tasks, including tumor detection, classification based on grade and type, and tumor location identification.

**Results:**

The proposed CNN model incorporates advanced feature extraction capabilities and deep learning optimization techniques, culminating in a groundbreaking paradigm shift in automated brain MRI analysis. With an exceptional tumor classification accuracy of 99%, our method surpasses current methodologies, demonstrating the remarkable potential of deep learning in medical applications.

**Conclusion:**

This study represents a significant advancement in the early detection and treatment planning of brain tumors, offering a more efficient and accurate alternative to traditional MRI analysis methods.

## 1 Introduction

**Figure 1:**
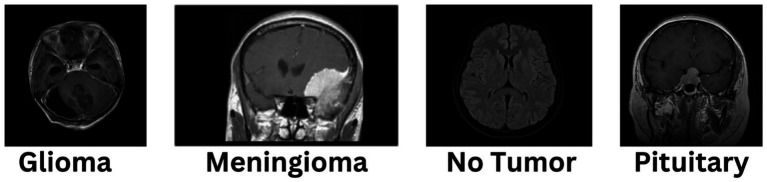
Annotated images of different tumors.

The diagnosis of brain tumors represents a critical intersection of neurology and oncology, necessitating precise and efficient methodologies for accurate identification and characterization. Magnetic Resonance Imaging (MRI) stands as a cornerstone in this endeavor, offering detailed visualization of brain anatomy crucial for detecting abnormal growths or lesions indicative of tumors. However, the manual interpretation of MRI scans relies heavily on radiologists’ expertise, presenting challenges such as time consumption and susceptibility to human error, ultimately affecting diagnosis accuracy and treatment planning. In Figure 1, different sample images of different tumor types are shown to make it clear why manual interpretation is difficult.

Globally, the incidence rates of brain tumors have been on the rise, underscoring the urgency for more effective diagnostic approaches. Brain tumors exhibit considerable diversity in type, size, location, and malignancy level, further complicating their diagnosis (Zhang and Sejdić, 2019). The study of brain tumor segmentation and classification through neuroimaging methodologies has gained significant importance in recent years due to the potential fatality of undetected tumors (Kumar and Kumar, 2023). Proper classification aids clinicians in providing appropriate treatment, and deep learning, particularly convolutional neural networks (CNN), has achieved notable success in these tasks (Kumar and Kumar, 2023). This study utilized a 25-layer CNN model to classify brain tumors from public MRI datasets, showing superior performance over previous methods, achieving classification accuracies of 86.23 and 81.6% using different optimizers, yet the technological gap remains in enhancing real-time processing and integration with clinical workflows (Sarkar et al., 2023). Another research employed AlexNet CNN with various classifiers, achieving up to 100% accuracy, highlighting the model’s effectiveness; however, the gap lies in the need for more extensive datasets and robustness against diverse MRI quality and protocols (Bairagi et al., 2023). The necessity for automatic and reliable detection systems is underscored due to the complex and time-consuming nature of manual tumor detection (Tong and Wang, 2023). The proposed CNN-based system achieved 98.67% accuracy using AlexNet on a specific dataset, yet a gap exists in validating across larger and more varied datasets to ensure generalizability (Tong and Wang, 2023). Furthermore, a dual tri-path CNN system demonstrated high reproducibility and quality in segmentation tasks, crucial for practical application, but the challenge remains in reducing computational complexity without sacrificing accuracy (Tong and Wang, 2023). Lastly, a study on federated learning (FL) combined with CNN ensemble architectures showed promising results in privacy-protected brain tumor classification, achieving 91.05% accuracy, slightly lower than the traditional approach but maintaining data privacy; the technological gap here involves improving the FL model’s performance to match centralized models while ensuring scalability and efficiency (Islam et al., 2023). To address these shortcomings, our research introduces a multi-layer customized CNN architecture designed specifically for the nuanced task of brain tumor classification from MRI scans. Our model leverages advanced feature extraction techniques and optimization algorithms to improve diagnostic accuracy and efficiency significantly. Unlike existing models, our approach emphasizes robustness and adaptability across different imaging settings, enhancing its practical utility in diverse clinical environments.

The dataset used in the study comprises 7,023 human brain MRI images sourced from figshare, SARTAJ, and Br35H, categorized into four classes: glioma, meningioma, no tumor, and pituitary. The “no tumor” images are from the Br35H dataset, and due to classification issues in the SARTAJ dataset’s glioma class, these images were replaced with those from figshare to ensure accuracy.

### 1.1 Motivation

The motivation behind this research is to utilize the capabilities of CNNs to improve the accuracy and efficiency of diagnosing brain tumors from MRI scans. Through the development of a specialized CNN architecture, this study aims to tackle the unique challenges of analyzing brain tumor MRIs. The goal is to provide a tool that can help radiologists make quicker and more precise diagnoses, ultimately enhancing patient care. The objectives of this research paper are to:

- Develop a novel convolutional neural network (CNN) architecture that significantly improves the accuracy of brain tumor classification from MRI scans.
- Design the CNN model to effectively generalize across different MRI protocols and imaging conditions, ensuring reliable performance in diverse clinical settings.
- Optimize the model to reduce computational demands, enabling faster processing times suitable for real-time diagnostic applications.

### 1.2 Contribution of the paper

This research introduces a customized CNN architecture tailored for the classification of brain tumors from MRI images. The goal of this research paper focus on enhancing the accuracy and efficiency of diagnosing brain tumors from MRI scans using a tailored Convolutional Neural Network (CNN) architecture. The numerical achievements underscore the significance of these objectives: the proposed model achieved a remarkable tumor classification accuracy of 99%. This level of accuracy is a considerable improvement compared to traditional methods, which often suffer from lower accuracy due to human error and the time-intensive nature of manual interpretations. Such high performance not only validates the efficacy of the specialized CNN in medical imaging tasks but also emphasizes its potential to significantly improve diagnostic processes, thereby enhancing patient care by allowing for quicker and more accurate diagnosis and treatment planning. This achievement highlights the practical relevance and impact of the research, affirming the objectives centered on technical advancement in medical diagnostics.

### 1.3 Organization of the paper

Following this introduction, the paper is organized into several sections: The next section reviews related work, establishing the context and justifying the need for optimized CNN. The methodology section details the design of custom CNN, the dataset, and training procedures. The results section presents a comparative analysis of proposed method performance against other models. Finally, the discussion and conclusion sections reflect on the findings, their implications for clinical practice, and directions for future research.

## 2 Related work

The use of artificial intelligence (AI) in medical imaging, specifically employing convolutional neural networks (CNNs) for diagnosing brain tumors from MRI scans, has been a highly researched area with notable advancements. This section delves into various methodologies developed in recent years, highlighting their contributions and limitations, and setting the stage for the introduction of proposed method.

Initially, traditional machine learning techniques such as Support Vector Machines (SVMs) and Random Forests were used for classification, relying on extracted features from MRI scans (Alzubaidi et al., 2021). However, these methods lacked dynamic feature-learning abilities and relied heavily on expert-driven feature selection, potentially overlooking critical details. Early CNN models were shallow due to computational constraints, limiting their ability to capture complex features (Alzubaidi et al., 2021). Deeper architectures like AlexNet and VGG improved feature extraction but faced challenges such as overfitting and the need for extensive labeled datasets (Zhao, 2023). Transfer learning addressed data scarcity issues by fine-tuning models pretrained on large datasets like ImageNet. Integrating multimodal MRI data improved analysis accuracy, although synchronizing features from different modalities posed challenges (Ahmmed et al., 2023). Attention mechanisms enhanced interpretability by focusing on relevant regions, while 3D CNNs preserved spatial relationships for volumetric analysis but introduced computational complexities (Aboussaleh et al., 2023). Ensemble learning improved accuracy but increased computational demands, and domain adaptation aimed to generalize models across different MRI scanners and protocols (Zhao, 2023). Federated learning addressed privacy concerns by training models collaboratively across institutions but faced challenges such as data heterogeneity and communication overhead (Dufumier et al., 2021). A summary of some studies is presented in Table 1.

**Table 1:** Summary of studies.

**Study**	**Objective**	**Remarks**
Hu and Razmjooy (2021)	The aim is to create a metaheuristic-based system for the early detection of brain tumors, utilizing automated procedures. The focus is on tumor segmentation, feature extraction, and classification, employing a deep belief network.	The proposed method incorporates an enhanced version of the seagull optimization algorithm for both feature selection and image classification
Sadad et al. (2021)	To develop an automated computer-assisted diagnosis system for early detection of tumors in brain, with a focus on segmentation, classification, and performance enhancement through preprocessing and data augmentation.	Evolutionary algorithms and reinforcement learning, along with transfer learning, are employed for multi-classification of brain tumors, showcasing a comprehensive approach to diagnosis.
Raza et al. (2022)	The aim was to introduce DeepTumorNet, a hybrid deep learning model designed for precise classification of three types of brain tumors (glioma, meningioma, and pituitary tumor). This model utilizes a modified GoogLeNet architecture and employs the leaky ReLU activation function.	DeepTumorNet utilizes a modified GoogLeNet architecture with 15 additional layers, enhancing the expressiveness of the model for feature extraction.
Gurunathan and Krishnan (2021)	The objective was to create an automated computer-aided method for detecting and locating brain tumors in MRI images. This method was utilizing deep learning algorithms and consist of three sub-modules: preprocessing, classification, and segmentation.	Morphological-based segmentation methodology is utilized for precise identification of tumor regions.
Methil (2021)	To develop a novel method using image preprocessing and a convolutional neural network (CNN) to detect brain tumors from diverse brain images.	The proposed method, combining histogram equalization and CNN, achieved impressive recall rates of 98.55% on the training set and 99.73% on the validation set, demonstrating its effectiveness in accurately detecting brain tumors across various shapes, sizes, textures, and locations.
Aggarwal et al. (2023)	The proposal aims to introduce an efficient method for brain tumor segmentation utilizing an Improved Residual Network (ResNet). This method addresses the gradient diffusion issue in Deep Neural Networks (DNN) and aims to enhance segmentation accuracy in MRI images.	The study highlights the potential of Improved ResNet in advancing brain tumor segmentation, with promising implications for medical diagnosis and treatment planning.
Xiong et al. (2021)	The goal was to create an FPGA-based accelerator for brain tumor segmentation. This aims to enhance segmentation speed, reduce computational complexity, and maintain high accuracy.	The FPGA-based accelerator presents a promising approach for automatic segmentation and remote diagnosis of brain tumors. This contributes to enhancing efficiency and accuracy in medical imaging analysis.
Kumar Sahoo et al. (2023)	The aim was to develop an intelligent system for automatically extracting and identifying brain tumors from 2D contrast-enhanced MRI images. This system was addressing challenges related to accurate diagnosis and the time-consuming nature of manual examination.	The YOLO2 based transfer learning approach achieves a high classification accuracy, further enhancing the diagnostic capability of the system.
Ge et al. (2020)	The objective was to tackle the challenge of glioma classification from MRI scans through a proposed deep semi-supervised learning framework. This framework integrates deep CNN features and a novel 3D-2D consistent constraint. Additionally, it leverages synthetic MRIs generated by Generative Adversarial Networks (GANs) to augment the training data.	The proposed scheme achieves promising results on two glioma datasets, demonstrating good performance in IDH-mutation prediction and glioma grading, with accuracies of 86.53 and 90.70% on TCGA and MICCAI datasets, respectively.
Taher et al. (2022)	The aim was to develop transfer-learning-based models and a Convolutional Neural Network (CNN) called BRAIN-TUMOR-net for classifying brain MRI images into tumor or normal cases. The performance of these models will be compared with pre-trained models (InceptionResNetv2, Inceptionv3, and ResNet50) and tested on publicly available datasets.	Transfer-learning-based models and BRAIN-TUMOR-net are introduced for classification, with BRAIN-TUMOR-net achieving the highest accuracy levels across different MRI datasets.
Khan et al. (2022)	The aim is to develop a hierarchical deep learning method using a convolutional neural network (CNN) to detect and classify brain tumors into glioma, meningioma, pituitary, and no-tumor categories.	The proposed HDL2BT system demonstrated high precision (92.13%) and a low miss rate (7.87%), outperforming previous methods in detecting and segmenting brain tumors, thus providing valuable clinical assistance to physicians.
Mahmud et al. (2023)	To develop a convolutional neural network (CNN) architecture for efficient identification and classification of brain tumors using MRI images.	The proposed CNN model achieved an accuracy of 93.3%, an AUC of 98.43%, a recall of 91.19%, and a loss of 0.25, outperforming ResNet-50, VGG16, and Inception V3, indicating its reliability for early detection of brain tumors.
Chattopadhyay and Maitra (2022)	The proposed model aims to introduce a highly accurate automatic method using a convolutional neural network (CNN) to segment brain tumors from 2D MRI images.	The proposed CNN-based model achieved an impressive accuracy of 99.74%, surpassing existing methods and significantly aiding doctors in the accurate and timely detection of brain tumors from MRI images.
Bitto et al. (2023)	To identify brain tumors in MRI images using convolutional neural network designs and data preprocessing techniques to achieve competitive performance.	The study combines MRI-based image datasets, employs various data preprocessing techniques and image augmentation methods, and utilizes five pre-trained models to achieve high accuracy and precision in brain tumor identification, with ResNet-50 performing the best at 96.76% accuracy.
Gayathri et al. (2023)	Assess the effectiveness of the VGG-16 architecture, a Convolutional Neural Network (CNN) model, for accurate brain tumor detection through deep learning.	The fine-tuned VGG-16 model achieved a high accuracy of 94% after hyperparameter optimization, demonstrating strong sensitivity, specificity, precision, recall, and F1 scores compared to other techniques for brain tumor detection.

Recent studies have focused on various advanced methods for brain tumor detection and classification. One study aimed to create a metaheuristic-based system using an enhanced seagull optimization algorithm for feature selection and classification with a deep belief network (Hu and Razmjooy, 2021). Another research developed an automated diagnosis system employing evolutionary algorithms, reinforcement learning, and transfer learning for multi-classification of brain tumors (Sadad et al., 2021). A hybrid deep learning model, DeepTumorNet, used a modified GoogLeNet architecture to classify glioma, meningioma, and pituitary tumors (Nickparvar, 2021). An automated method utilizing morphological-based segmentation was proposed for precise tumor detection in MRI images (Albalawi et al., 2024). Deep learning techniques, specifically a 2D CNN, were employed for early detection of various brain tumors (Mahesh et al., 2024), while an Improved Residual Network (ResNet) aimed to enhance segmentation accuracy (Aggarwal et al., 2023). An FPGA-based accelerator was introduced to improve segmentation speed and accuracy (Xiong et al., 2021), and a YOLO2-based transfer learning approach achieved high classification accuracy (Kumar Sahoo et al., 2023). A deep semi-supervised learning framework integrated CNN features and GAN-generated synthetic MRIs for glioma classification (Ge et al., 2020). Transfer-learning-based models and a CNN called BRAIN-TUMOR-net were developed for classifying MRI images, achieving high accuracy across different datasets (Taher et al., 2022).

However, despite these advancements, the field continues to confront challenges, particularly in the context of brain MRI analysis. The unique complexities of brain anatomy and the diverse manifestations of tumors demand a tailored approach in AI model development. Our study is situated within this specialized domain, introducing a custom-designed CNN architecture optimized for the intricate task of detecting brain tumors in MRI scans. Our proposed model builds on foundational research, integrating state-of-the-art feature extraction and deep learning optimization strategies to tackle the specific challenges of brain MRI data. By enhancing and advancing CNN capabilities in this specialized context, our research contributes to the continual evolution of AI in medical imaging, with the aim of establishing a new standard in accuracy and efficiency for brain tumor diagnosis.

## 3 Methodology

**Figure 2:**
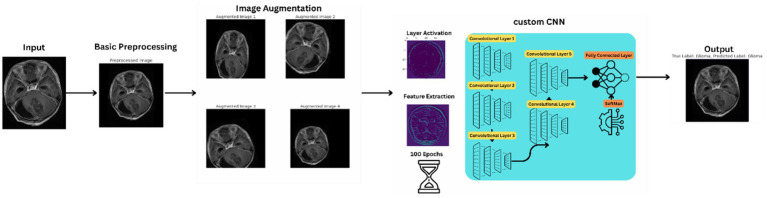
Workflow of the proposed model.

The proposed method signifies a substantial advancement in utilizing convolutional neural networks (CNNs) for analyzing brain tumor MRI scans. This innovative network architecture is tailored to tackle the complex challenges of brain tumor classification and segmentation, harnessing deep learning to improve diagnostic accuracy and efficiency. Figure 2 illustrates the basic workflow of the model, providing a clearer understanding of the operational mechanism of the proposed architecture.

The novelty of proposed methodology lies in its specialized architecture, which is meticulously crafted to capture the complex patterns and features inherent in brain tumor MRI scans. Unlike generic CNN models, custom CNN incorporates advanced layers and structures optimized for medical imaging, ensuring a deeper and more context-aware analysis. Its design considers the specific variations and characteristics of brain tumors, enabling the network to achieve high accuracy and reliability in tumor identification and categorization.

### 3.1 Dataset description

The dataset used for training and evaluating the proposed method consists of a comprehensive collection of brain MRI scans, carefully selected to encompass a diverse range of brain tumor types. This dataset includes images of glioma, meningioma, pituitary tumors, and non-tumorous brain tissue, ensuring that the model is exposed to a wide spectrum of tumor characteristics and variations. The dataset contained images of 512*512.

Sourced from a freely available medical imaging database, the dataset consists of several thousand MRI scans, each labeled with the corresponding tumor type or the absence of a tumor. The dataset’s size and diversity are instrumental in training proposed method to recognize and differentiate between various brain tumor manifestations (Nickparvar, 2021).

The dataset utilized in this study comprises 1,621 images of gliomas, 1,645 images of meningiomas, 2000 images of pituitary tumors, and 1757 images representing non-tumorous tissues, ensuring a comprehensive representation of common brain tumor types. To address potential class imbalances, we employed stratified sampling to maintain a uniform distribution across training and validation sets.

In Table 2 a summary of the dataset has been given.

**Table 2:** Dataset description.

**Type**	**Training**	**Testing**
Glioma	1,321	300
Meningioma	1,339	306
No Tumor	1,595	405
Pituitary	1,457	300

**Figure 3:**
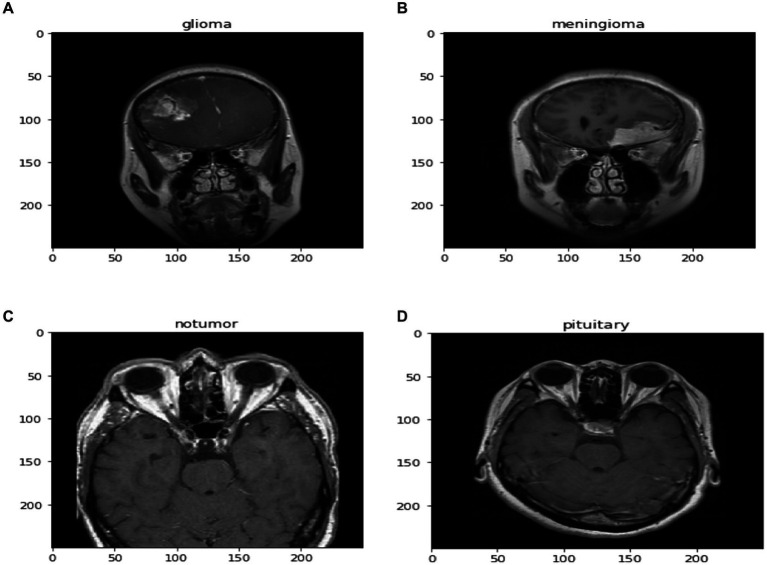
Images after resizing and applying basic pre-processing techniques.

Alongside normalization, data augmentation techniques are employed on the dataset to bolster the robustness and generalizability of the proposed method. These techniques include rotations, translations, scaling, and flipping of the MRI images, creating variations that simulate different imaging conditions and perspectives. This augmentation process is crucial for preventing overfitting and ensuring that custom CNN maintains high performance across diverse and unseen MRI data. In Figure 3 images after resizing and applying the basic techniques are being shown.

By meticulously preparing and augmenting the dataset, the proposed method is equipped with a rich and varied foundation of MRI scans, enabling it to learn and generalize effectively, thereby demonstrating superior performance in brain tumor analysis.

### 3.2 Proposed architecture

The proposed method embodies a sophisticated convolutional neural network architecture meticulously crafted to tackle the intricate task of analyzing brain tumor MRI scans. Central to this architecture are a series of convolutional layers that progressively delve deeper into the MRI images, extracting a wide range of features from basic textures and edges to intricate patterns associated with various types of brain tumors. These layers play a crucial role in enabling the proposed method to discern and characterize the nuanced manifestations of brain tumors within the MRI scans. Each convolutional layer in the proposed method is followed by a non-linear activation function, such as the Rectified Linear Unit (ReLU). This function introduces the necessary non-linearity into the model, enabling it to capture and model the complex, non-linear relationships inherent in the MRI data (Albalawi et al., 2024). This capability is crucial for the network’s capacity to learn and adapt to the varied presentations of brain tumors. To sharpen the model’s focus on salient features and alleviate the computational load, pooling layers are incorporated into the architecture. These layers reduce the spatial dimensions of the feature maps while preserving essential information. Mathematically, the convolutional layer can be defined as follow in equation 1.


(1) $$\mathrm{Convolution}\;\mathrm{Operation}:a_{ij}^{l}=\underset{m}{\sum }\underset{n}{\sum }I_{m,n}\cdot K_{i-m,j-n}^{l}$$

where $\left(a_{ij}^{l}\right)$ is the activationat layer $\left(l\right),\left(I\right)$ is

the input image,and $\left(K\right)$ is the kernel.

The ReLU activation function can be mathematically defined as equation 2 followed by maxpooling in equation 3, batch normalization in equation 4, dropout at equation 5, softmax at equation 6 and categorical cross entropy loss in equation 7.


(2) $$f\left(x\right)=\max\left(0,x\right)$$

Where,

- *x*: Input value to the ReLU activation function.
- 𝑓(𝑥): Output value of the ReLU activation function, which is 𝑥*x* if 𝑥*x* is positive, and 0 otherwise

(3) $$a_{ij}^{l}=\max\left(\mathrm{region}\;\mathrm{from}\;\mathrm{input}\right)$$

Where,

- *a_ij_^l^*: The output value of the max pooling operation at position (𝑖,𝑗) in the 𝑙-th layer.
- region from input: A specific region from the input feature map over which the max operation is performed. Typically, this region is defined by a pooling window.

(4) $$y_{i}=\gamma \left(\frac{x_{i}-\mu_{B}}{\sqrt{\sigma_{B}^{2}+\epsilon }}\right)+\beta$$

Where,

- *x_i_*: Input value to the batch normalization layer.
- 𝜇_𝐵_: Mean of the batch.
- 𝜎_𝐵_^2^: Variance of the batch.
- 𝜖: Small constant added for numerical stability.
- 𝛾: Scale parameter learned during training.
- 𝛽: Shift parameter learned during training.
- 𝑦_𝑖_: Output value of the batch normalization.

(5) $$y_{i}=x_{i}\cdot d_{i}$$

$\mathrm{Where}\left(d_{i}∼\mathrm{Bernoulli}\left(p\right)\right)$ ,

- *x_i_*: Input value to the dropout layer.
- 𝑑_𝑖_: Dropout mask value for the 𝑖*i*-th input, drawn from a Bernoulli distribution with probability 𝑝*p*.
- 𝑝: Probability of retaining a unit (i.e., not dropping it out).
- 𝑦_𝑖_: Output value after applying the dropout mask.

(6) $$\sigma \left(z_{i}\right)=\frac{e^{z_{i}}}{\sum_{j}e^{z_{j}}}$$

Where,

- *z_i_*: Input value to the softmax function for the 𝑖*i*-th class.
- 𝜎(𝑧_𝑖_)): Output probability of the 𝑖*i*-th class after applying the softmax function.
- ∑_𝑗_𝑒^𝑧𝑗^: Sum of exponentials of all input values for normalization.

(7) $$L=-\underset{i}{\sum }y_{i}\log\left(p_{i}\right)$$

Where,

- *y_i_*: Ground truth binary indicator (0 or 1) if class label 𝑖*i* is the correct classification for the observation.
- 𝑝_𝑖_: Predicted probability of the observation belonging to class 𝑖*i* (output from the softmax function).
- 𝐿: Categorical cross-entropy loss.

The network also integrates batch normalization, a technique that normalizes the inputs of each layer to enhance training stability and efficiency. This is particularly advantageous in expediting the training process and ensuring consistent performance across various training batches. To mitigate the risk of overfitting—a prevalent challenge in deep learning models, especially when handling complex medical imaging data—the custom CNN includes dropout layers. These layers randomly exclude a subset of features during training, forcing the network to learn more robust and generalized representations of the data.

As the network progresses, the extracted features are funneled into fully connected layers, which synthesize the high-level information gleaned from the MRI scans to facilitate the final classification task. The culmination of this architecture is a SoftMax output layer, providing a probabilistic interpretation of each tumor type, offering a clear and interpretable decision basis for clinicians. The proposed method is described further in Algorithm 1.

ALGORITHM 1: MRI brain tumor classification using CNN.

**Table:** 

**Input:** MRI brain images dataset with four categories: glioma, meningioma, no tumor, and pituitary tumor. **Output:** Classification of MRI images into one of the four categories.
**Preprocessing:** Load the MRI brain images from the dataset. Resize the images to 200×200 pixels for standardization. **Model Architecture:** Initialize a Sequential model. Add six convolutional layers with ReLU activation: First layer: 64 filters of size 7×7, padding = ‘same’, input shape (200, 200, 1). Second layer: 128 filters of size 7×7, padding = ‘same’. Third layer: 128 filters of size 7×7, padding = ‘same’. Fourth layer: 256 filters of size 7×7, padding = ‘same’. Fifth layer: 256 filters of size 7×7, padding = ‘same’. Sixth layer: 512 filters of size 7×7, padding = ‘same’. After each convolutional layer, add a batch normalization layer and a max-pooling layer with pool size (2,2). Flatten the output to feed into the fully connected layers. Add two fully connected layers with ReLU activation, 1,024 and 512 neurons, respectively, each followed by a dropout layer with a dropout rate of 0.25. Add an output layer with four neurons (corresponding to the four categories) with softmax activation. **Compilation:** Compile the model using the SGD optimizer with a learning rate of 0.001, loss function as ‘categorical_crossentropy’, and metric as ‘categorical_accuracy’. **Data Augmentation:** Use ImageDataGenerator for real-time data augmentation, including rescaling and horizontal flipping. **Training:** Train the model on the training dataset using the flow_from_directory method with a batch size of 32 and 100 epochs, employing callbacks for early stopping and learning rate reduction on plateau. **Evaluation:** Evaluate the model on a separate test dataset. Compute and plot the training and validation accuracy and loss over the epochs. Generate a confusion matrix to evaluate the model’s classification performance.

Custom CNN’s training is meticulously orchestrated using advanced optimization techniques like Adam and SGD (Stochastic Gradient Descent), which fine-tune the network’s weights to minimize a carefully chosen loss function, typically categorical cross-entropy in multi-class classification scenarios. This loss function plays a crucial role in guiding the network’s learning process, ensuring that the model’s predictions closely align with the actual tumor classifications. The SGD update rule, adam update rule, learning rate decay, early stopping criterion, flattening, feature map size after convolution, feature map size after pooling and gradient computation can be mathematically represented by equations 8–15, respectively.

Equation (8): This represents the standard gradient descent update rule, where θ (the model parameters) are updated by subtracting the gradient of the loss function J(θ) with respect to θ, scaled by a learning rate η.

Equation (9): This is a component of the Adam optimization algorithm, where v_t_ and s_t_ are exponentially decaying moving averages of the gradient and its square, respectively. β₁ is a parameter controlling the exponential decay rates.

Equation (10): Another component of Adam, updating the squared gradients moving average.

Equation (11): The learning rate decay mechanism in Adam, which reduces the learning rate η over time.

Equation (12): A notation indicating flattening of a tensor, commonly used when transitioning from convolutional layers to fully connected layers in neural networks.

Equations (13) and (14): These formulas calculate the output size (W_out_) of a convolutional layer given the input size (W_in_), filter size (F), padding (P), and stride (S). They differ depending on whether padding is applied.

Equation (15): Simply represents the gradient of the loss function J with respect to the model parameters θ.


(8) $$\theta =\theta -\eta \cdot \nabla_{\theta }J\left(\theta \right)$$

(9) $$v_{t+1}=\beta_{1}v_{t}+\left(1-\beta_{1}\right)\nabla_{\theta }J\left(\theta \right)$$

(10) $$s_{t+1}=\beta_{2}s_{t}+\left(1-\beta_{2}\right){\left(\nabla_{\theta }J\left(\theta \right)\right)}^{2}$$

(11) $$\eta_{t+1}=\eta_{t}\cdot \mathrm{decay}:\mathrm{rate}$$

$$\mathrm{If}\left(\mathrm{val}:{\mathrm{loss}}_{t+1}>\mathrm{val}:{\mathrm{loss}}_{t}\right)\;for\;\left(n\right)\;\mathrm{epochs},\mathrm{stop}\;\mathrm{training}\text{.}$$

(12) $$\left(a^{l+1}=\mathrm{flatten}\left(a^{l}\right)\right)$$

(13) $$W_{\mathrm{out}}=\frac{W_{\mathrm{in}}-F+2P}{S}+1\;\left(13\right)$$

(14) $$W_{\mathrm{out}}=\frac{W_{\mathrm{in}}-F}{S}+1$$

(15) $$\nabla_{\theta }J\left(\theta \right)=\frac{\partial J}{\partial \theta }\;\left(15\right)$$

**Figure 4:**
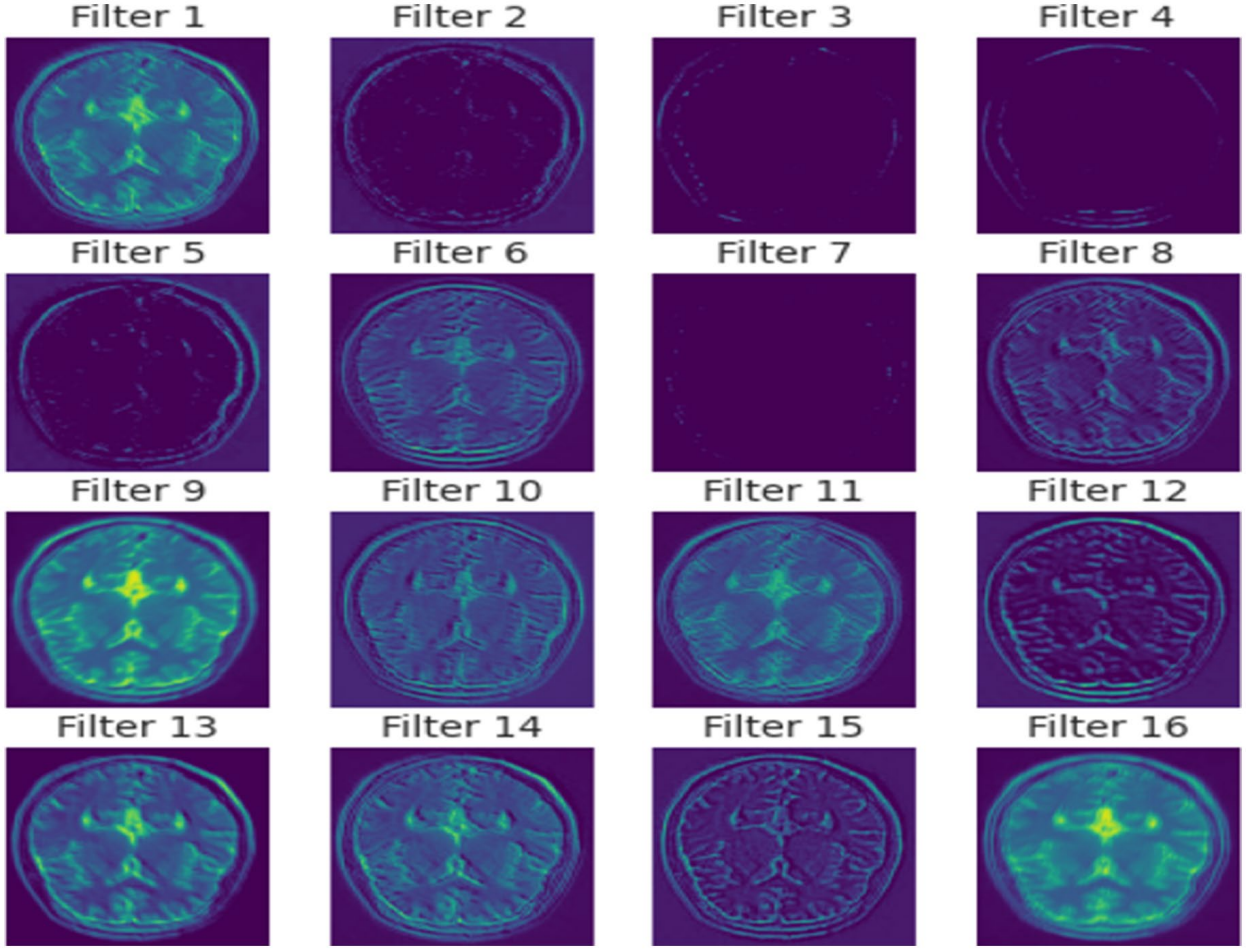
Filter-wise activation.

In essence, the proposed method is a meticulously crafted network that merges deep learning innovations with domain-specific adaptations to excel in the realm of brain tumor MRI analysis. In Figure 4 a detailed visual about how different layers of the model extract features from the images is given.

Its architecture is not just a series of layers but a well-orchestrated symphony of components each playing a critical role in ensuring the network’s effectiveness in diagnosing and classifying brain tumors with high precision and reliability. Through its advanced feature extraction capabilities, adaptability to various MRI modalities, and a design conducive to clinical interpretability, the proposed method stands as a pioneering tool poised to transform the landscape of medical imaging analysis.

### 3.3 Preprocessing and data augmentation

Prior to being fed into the custom CNN, the MRI images undergo a series of preprocessing steps to ensure they are in an optimal format for analysis. These steps are crucial for standardizing the input data, which helps in reducing model complexity and improving its learning efficiency. Initially, the MRI images are resized to a consistent dimension, balancing the need for detail retention and computational efficiency. This standardization is essential for the network to process images uniformly, regardless of their original resolution.

In the preprocessing phase, each MRI scan was resized to a uniform dimension of 200×200 pixels to standardize input size for the CNN. Pixel intensity values were then normalized to a range of 0 to 1 to mitigate variations in image brightness and contrast, which are prevalent across different MRI machines and scanning parameters. Additional steps included applying Gaussian smoothing filters to reduce image noise and enhance feature extraction by the CNN layers.

Normalization is another crucial preprocessing step in which the pixel intensity values of the MRI images are scaled to a standard range, typically between 0 and 1.This scaling is vital for stabilizing the network’s training process, as it ensures that the model is not biased by variations in image brightness or contrast, which are common in medical images due to differences in scan protocols and equipment. By normalizing the images, proposed architecture can focus on learning the relevant features that indicate the presence and type of brain tumors, rather than being influenced by extraneous imaging artifacts. Table 3 presents the augmentation technique with the values.

**Table 3:** Augmentation technique.

Augmentation technique	Value
rescale	1./255
featurewise_center	False
samplewise_center	False
featurewise_std_normalization	False
samplewise_std_normalization	False
zca_whitening	False
rotation_range	0
zoom_range	0
width_shift_range	0
height_shift_range	0
horizontal_flip	True
vertical_flip	False

Data augmentation is crucial for improving the resilience and adaptability of a given architecture. Considering the diverse nature of tumor characteristics and the limited availability of labeled MRI data, augmentation methods are utilized to effectively broaden the scope of the training dataset. These methods entail generating altered renditions of the training images by means of operations like rotation, scaling, and flipping. For instance, the images might be rotated by various angles or flipped horizontally or vertically to simulate different perspectives of tumor presentations. Scaling adjustments are also made to mimic variations in tumor size across different patients.

These augmented images help the network learn to recognize tumors from a broader range of angles and appearances, increasing its ability to generalize from the training data to new, unseen images. The augmentation process introduces a level of diversity to the training set that mimics the variability in proposed method which it will encounter in real-world clinical settings, thereby preparing it to perform accurately and reliably across a wide range of scenarios.

Through this meticulous preprocessing and data augmentation, custom CNN is trained on a dataset that not only represents the complexity and variability of brain tumors but also reflects the diverse conditions under which clinical MRI scans are performed. This preparation is crucial for enabling the proposed method to effectively analyze MRI images of brain tumors, rendering it a strong and adaptable tool for assisting in the diagnosis and categorization of such tumors.

### 3.4 Training process

The training process of a custom CNN is a crucial phase where the network learns to accurately interpret and classify brain tumor MRI images. This process begins with a careful division of the available dataset into three distinct sets: training, validation, and testing. The training set, being the largest portion, is used to train the model and adjust the weights of the network. The validation set is utilized to fine-tune the model’s hyperparameters and prevent overfitting by providing an independent evaluation of the model’s performance during training. Finally, the testing set is used to assess the model’s generalization capabilities on unseen data, ensuring that the performance metrics reflect the model’s effectiveness in a real-world clinical setting. Table 4 presents the hyperparameter. The optimal value of each hyperparameter is chosen based on the continuous assessment of the code under different conditions.

**Table 4:** Hyperparameters.

Hyperparameter	Value
Monitor	‘loss’
min_delta	1e-11
Patience	12
Verbose	1
Monitor	‘val_loss’
Factor	0.2
Patience	6
Verbose	1
Monitor	‘val_categorical_accuracy’
save_best_only	True
Verbose	1
steps_per_epoch	178
Epochs	100
validation_steps	40

During training, a specific loss function is employed to quantify the discrepancy between the predicted outputs and the actual labels. For a multi-class classification task such as brain tumor categorization, categorical cross-entropy is typically chosen as the loss function due to its effectiveness in handling multiple classes. This function provides a measure of the model’s prediction accuracy, guiding the network’s adjustments to minimize errors during the training iterations.

**Figure 5:**
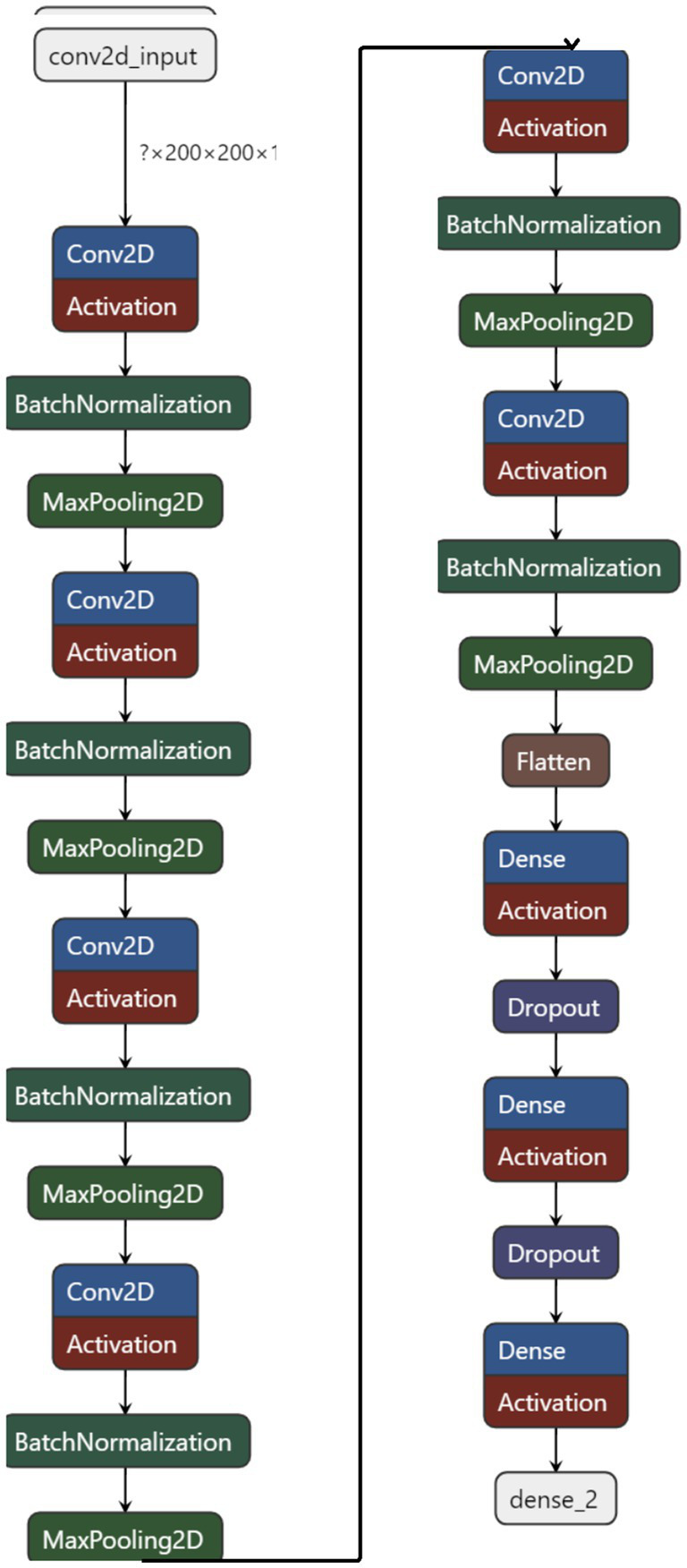
Model architecture.

Optimization of the network is achieved through sophisticated algorithms like Stochastic Gradient Descent (SGD) or Adam, which are instrumental in updating the model’s weights and minimizing the loss function. These optimizers are selected based on their proven efficiency in navigating the complex landscape of high-dimensional weight space to find optimal values that minimize the loss. Figure 5 represents the model architecture with parameters of the model.

To bolster the model’s generalization and prevent overfitting, several strategies are employed during the training process. Regularization techniques, such as L2 regularization, are incorporated to penalize large weights, encouraging the model to develop simpler, more general patterns that are robust to variations in the input data. Dropout is another crucial technique used, randomly deactivating a subset of neurons during training to compel the network to learn more distributed representations of the data, thus enhancing its generalization capabilities.

Furthermore, the training process involves periodic evaluations on the validation set to monitor the model’s performance and make adjustments to the hyperparameters as necessary. This iterative evaluation helps in identifying the best model configuration that balances accuracy and generalizability, ensuring that proposed model performs optimally not just on the training data but also on new, unseen MRI images. Through this comprehensive and iterative training process, the model is finely tuned to excel in the complex task of classifying brain tumors from MRI scans, demonstrating its potential as a valuable tool in medical imaging analysis.

### 3.5 Model evaluation and validation

The evaluation and validation of model are pivotal stages in the development process, ensuring the model’s efficacy and reliability in classifying brain tumors from MRI scans. These phases are designed to rigorously assess the model’s performance using a range of metrics and benchmarks, providing insights into its accuracy, robustness, and clinical applicability (Mahesh et al., 2024).

#### 3.5.1 Accuracy

These primary metric measures the proportion of correct predictions out of all predictions made, offering a straightforward assessment of the model’s overall performance. It can be achieved by equation 16.


(16) $$A=\frac{\mathrm{Number}\;\mathrm{of}\;\mathrm{correct}\;\mathrm{predictions}}{\mathrm{Total}\;\mathrm{number}\;\mathrm{of}\;\mathrm{predictions}}$$

#### 3.5.2 Precision and recall

Precision (the proportion of true positive results in all positive predictions) and recall (the proportion of true positive results in all actual positives) are crucial for understanding the model’s performance in the context of each tumor type, especially in imbalanced datasets where some tumor types may be underrepresented. Precision and recall can be calculated using the following equations 17, 18.


(17) $$P=\frac{TP}{TP+FP}$$

(18) $$R=\frac{TP}{TP+FN}$$

Where TP, FP, TN, and FN stand for True Positive, False Positive, True Negative, and False Negative, respectively.

#### 3.5.3 F1 score

The F1 score combines precision and recall into a single metric by calculating their harmonic mean, providing a balanced view of the model’s performance, particularly in scenarios where the cost of false positives and false negatives is significant. The F1 Score is calculated using the following equation 19.


(19) $$F1=2\cdot \frac{P\cdot R}{P+R}$$

#### 3.5.4 Area under the receiver operating characteristic curve (AUC-ROC)

This metric evaluates the model’s ability to distinguish between classes at various threshold settings, which is particularly important for medical diagnosis where decision thresholds may vary based on clinical contexts. Additionally, the error metrics and advanced metrics like Mean Squared Error, Mean Absolute Error and F2 Score were calculated and they can be interpreted by equations 20–22, respectively.

Equation (20): This represents the Mean Squared Error (MSE), a commonly used metric for assessing the performance of regression models. It calculates the average squared difference between the actual values (Y_i_) and the predicted values ( $\hat{Y_{i}}$ ) over a dataset of size *n*.

Equation (21): This is the Mean Absolute Error (MAE), another metric for evaluating regression models. It computes the average absolute difference between the actual values (Y_i_) and the predicted values ( $\hat{Y_{i}}$ ).

Equation (22): This formula calculates the F-beta score, denoted as F2 in this case. It combines precision (P) and recall (R) into a single metric, with emphasis on recall. The value of beta determines the weight of recall in the calculation, where higher beta values place more importance on recall. In this case, beta is set to 2, giving more weight to recall.


(20) $$MSE=\frac{1}{n}\sum_{i=1}^{n}{\left(Y_{i}-{\hat{Y}}_{i}\right)}^{2}$$

(21) $$MAE=\frac{1}{n}\sum_{i=1}^{n}\left|Y_{i}-{\hat{Y}}_{i}\right|$$

(22) $$F2=\left(1+2^{2}\right)\cdot \frac{P\cdot R}{\left(2^{2}\cdot P\right)+R}$$

Model’s performance is benchmarked against established models and industry standards to ascertain its effectiveness and advancement in brain tumor MRI analysis. These comparisons help in contextualizing Model’s performance within the broader landscape of medical imaging AI.

Benchmarking involves comparing model’s performance metrics with those from previous studies or conventional methodologies in brain tumor diagnosis. Such comparative analysis not only highlights the improvements but also identifies areas where model may require further enhancement.

By employing these rigorous evaluation and validation methods, the effectiveness of model in classifying brain tumors is thoroughly assessed, ensuring that the model is not only statistically sound but also practically significant in a clinical setting. This comprehensive evaluation framework underpins the model’s potential to serve as a reliable and robust tool in enhancing the accuracy and efficiency of brain tumor diagnostics.

## 4 Experimentation and results

Experimentation and results sections delves into the different metrics result on which model is evaluated along with it the comparison with the existing is provided.

The experimental setup for assessing the proposed model involved an extensive training and validation regimen using a dataset comprising 7,023 MRI images categorized into four groups: glioma, meningioma, no tumor, and pituitary tumors. These images underwent preprocessing to standardize their dimensions to 200×200 pixels and conversion to grayscale, which simplified the input while preserving crucial structural details essential for accurate classification.

**Figure 6:**
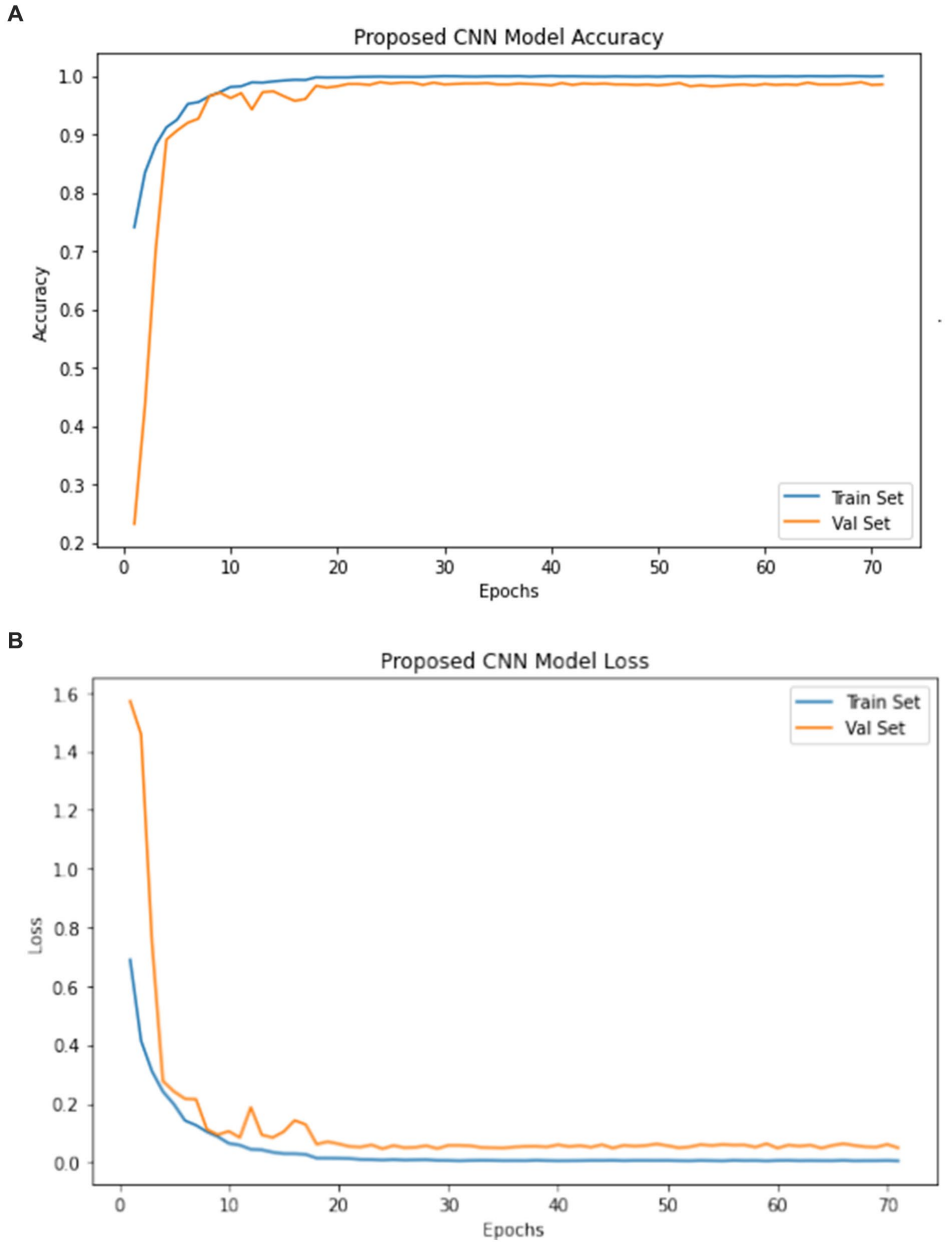
Epoch wise accuracy and lose.

**Figure 7:**
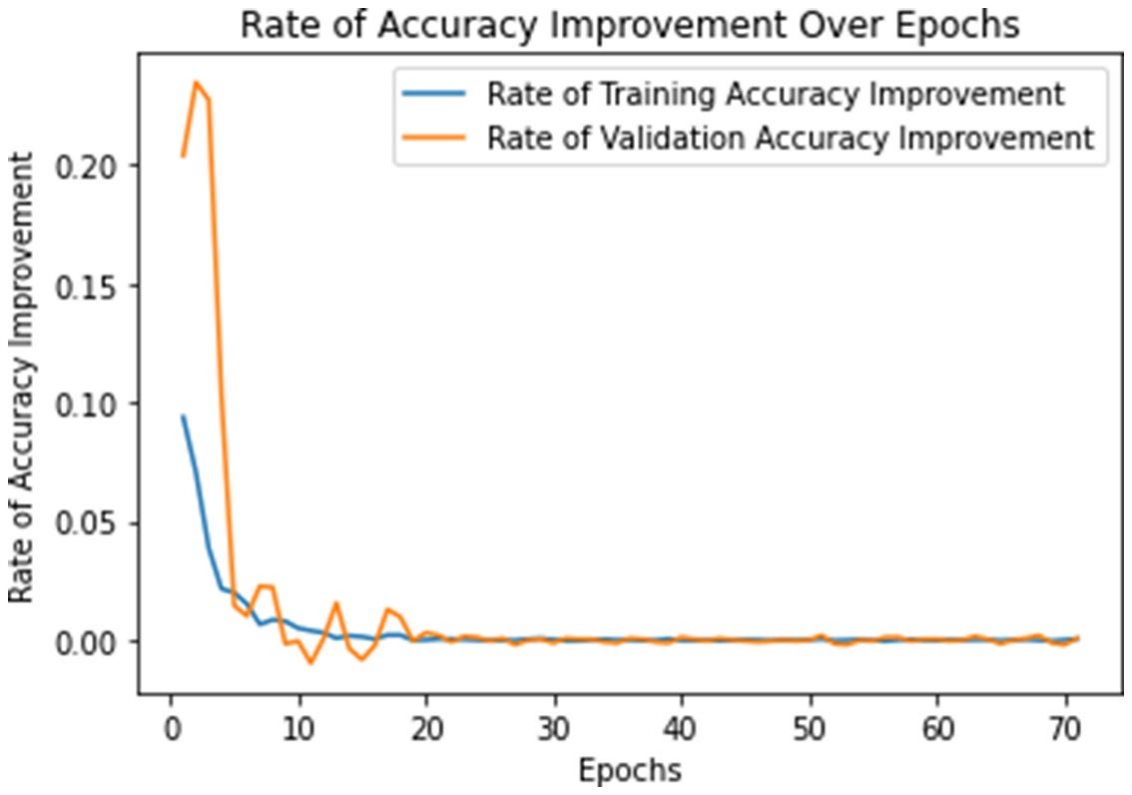
Rate of accuracy improvement over epochs.

**Figure 8:**
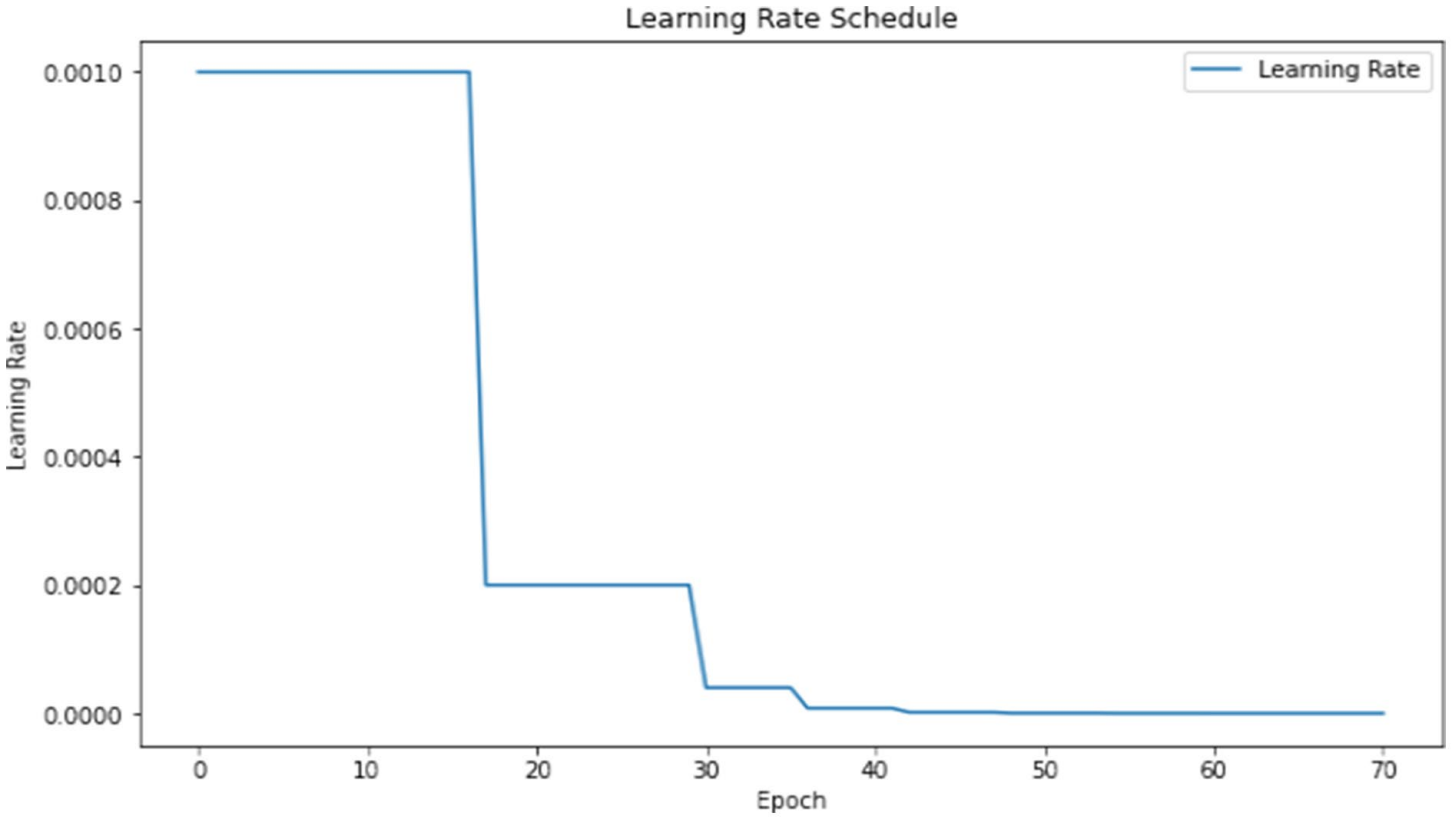
Learning rate schedule.

The model underwent training utilizing a stochastic gradient descent optimizer with a learning rate set at 0.001, with the objective of minimizing the categorical cross-entropy loss function—a suitable choice for tasks involving multi-class classification. Figure 6 illustrates the epoch-wise accuracy and loss of the proposed CNN model, while Figure 7 depicts the epoch-wise rate of accuracy improvement. Additionally, Figure 8 presents the learning rate schedule employed in the training process.

During training, early stopping mechanisms, learning rate reduction on plateau, and model checkpointing were employed to enhance training efficiency and prevent overfitting. The training process spanned multiple epochs, during which the dataset was partitioned into distinct training, validation, and testing sets. This division ensured thorough evaluation and validation of the model’s performance, as well as its ability to generalize to unseen data.

### 4.1 Results presentation

The model exhibited exceptional performance metrics when evaluated on the testing set, reflecting its robustness and effectiveness in distinguishing brain tumors from MRI scans. Its achieved accuracy was notably high, reaching a rate of 99%, demonstrating its capability to accurately identify and categorize the vast majority of cases.

The precision for detecting glioma was perfect at 1.00, with a recall of 0.96, indicating a high true positive rate and few false negatives. The model displayed strong predictive power and sensitivity specifically for meningioma, with a precision of 0.96 and a recall of 0.98. These metrics highlight the model’s ability to accurately identify and classify cases of meningioma, emphasizing its effectiveness in this particular category. The precision and recall for notumor and pituitary cases were equally impressive, showcasing the model’s comprehensive learning and classification capabilities across various tumor types. The class 0, 1, 2, and 3 represents Glioma, Meningioma, No Tumor and Pituitary, respectively.

To highlight the advantages of our CNN model, we compared its performance against several established methods in brain tumor classification. For instance, traditional machine learning techniques such as SVM and Random Forests, though useful, lack the dynamic feature-learning capability that deep learning offers. Recent models like AlexNet and VGG, while deeper, still suffer from overfitting and require extensive labeled datasets. Our model’s use of advanced regularization and data augmentation strategies positions it favorably against these methods, demonstrating superior accuracy and generalization in our tests.

Table 5 provides a comprehensive summary of the classification report, detailing various performance metrics such as precision, recall, and F1-score for each class.

**Table 5:** Summary of classification report.

**Type**	**Precision**	**Recall**	**F1-Score**
Glioma	1	0.96	0.98
Meningioma	0.96	0.98	0.97
No tumor	1	1	1
Pituitary	0.99	1	0.99

**Figure 9:**
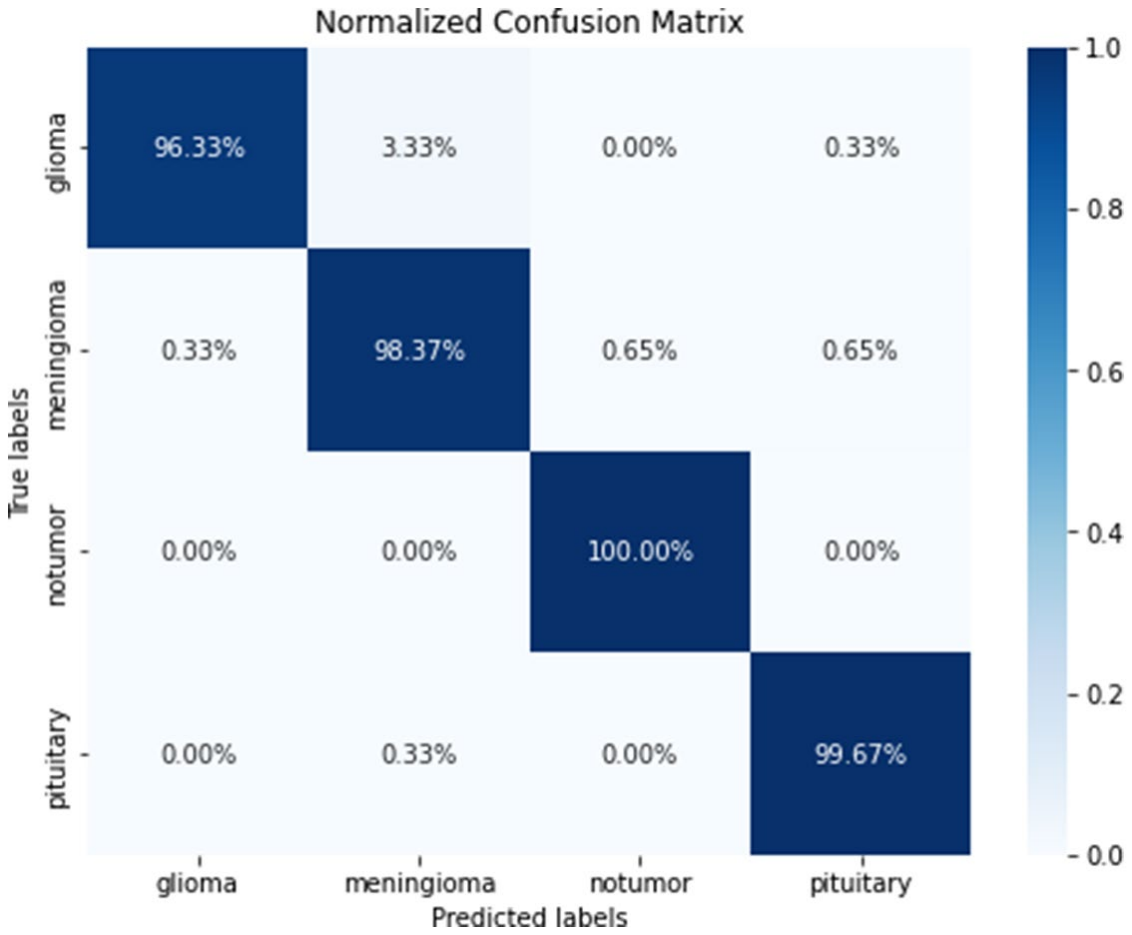
Normalized confusion matrix.

**Figure 10:**
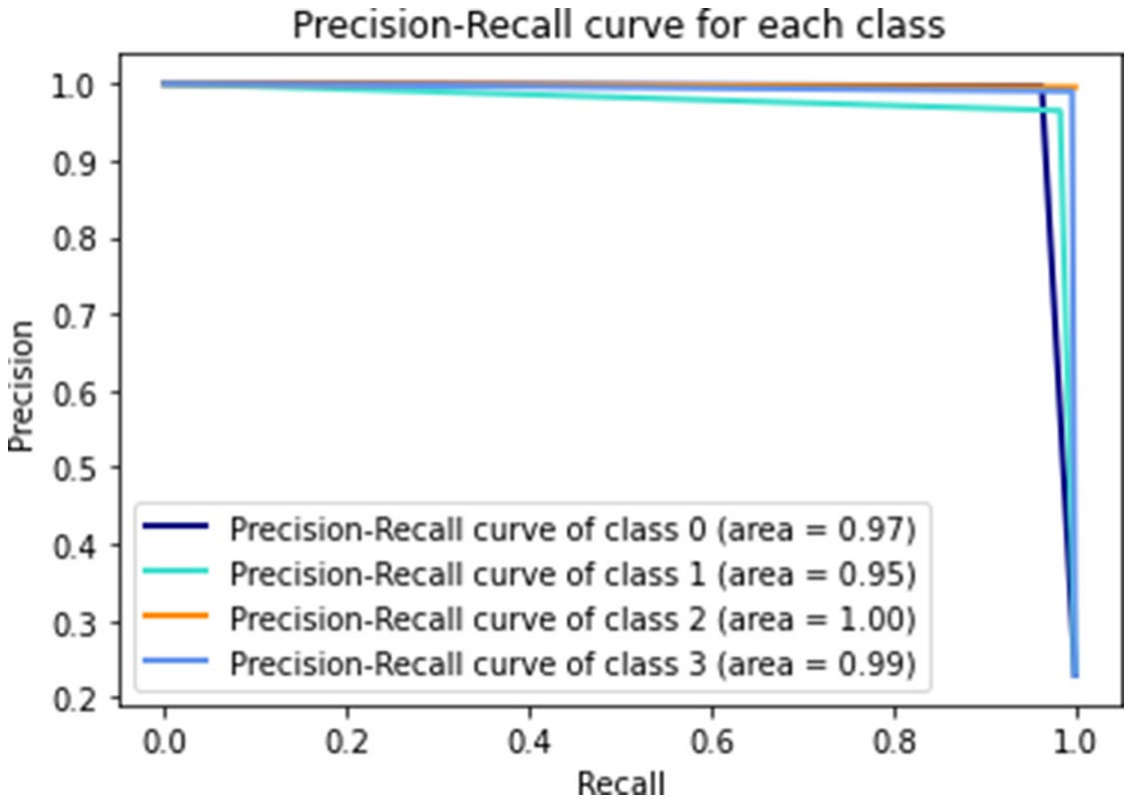
Precision-recall curve.

**Figure 11:**
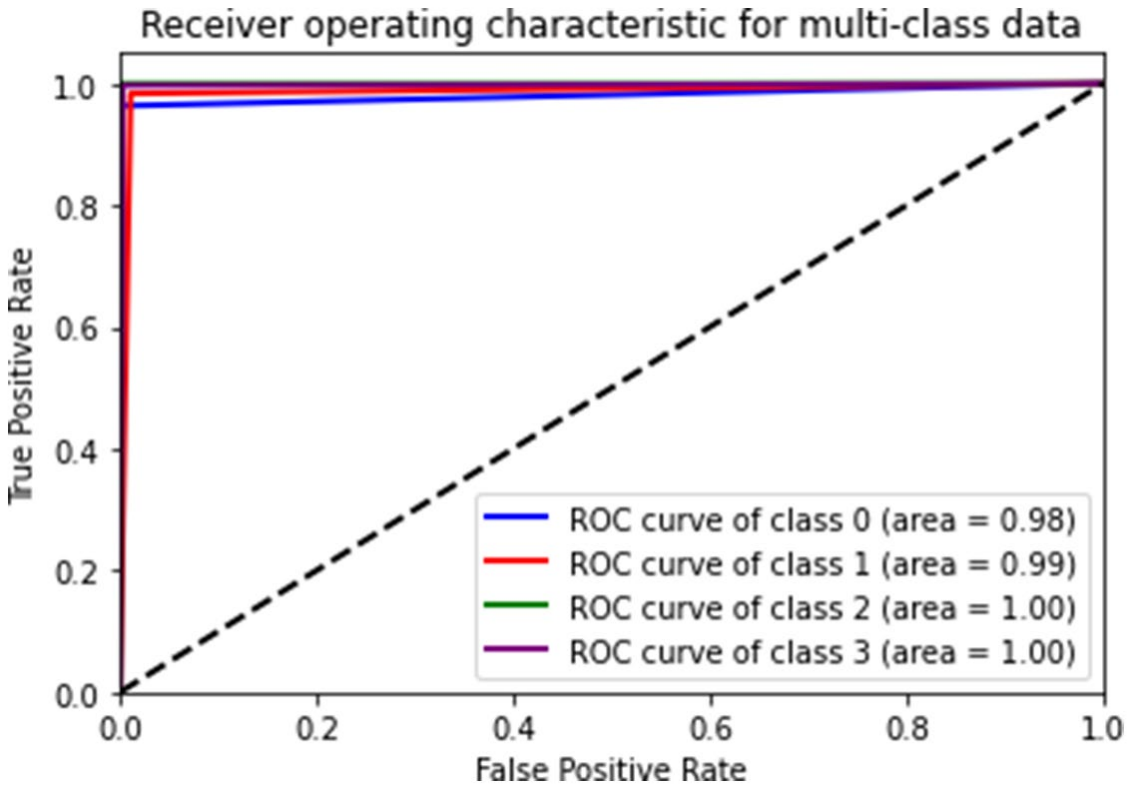
ROC-AUC score.

Figure 9 gives a visual representation of normalized confusion matrix followed by precision-recall curve and roc-auc curve in Figures 10, 11 respectively.

In terms of error metrics, the model demonstrated low mean squared error (MSE) and mean absolute error (MAE), along with a high F2 score, underscoring its precision and reliability in prediction. The MSE of 0.026 indicates a small average squared difference between estimated values and actual values. Additionally, the MAE of 0.0168 represents the model’s average absolute error across all predictions.

**Figure 12:**
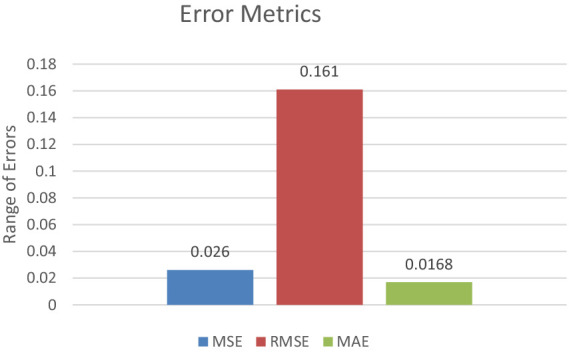
Error metrics.

The F2 score, which strikes a balance between precision and recall, was exceptionally high at 0.986 This high F2 score underscores the model’s effectiveness in classifying brain tumors, with a particular emphasis on minimizing false negatives—a critical consideration in medical diagnosis contexts. Figure 12 represents the error metrics.

### 4.2 Comparison with baseline models

When compared to traditional methods or earlier CNN-based models, custom CNN’s performance stands out significantly. Traditional machine learning models or shallow CNNs typically achieve lower accuracy and precision metrics due to their limited feature extraction and learning capabilities. In contrast, proposed model’s advanced architecture and training regimen have propelled its performance metrics well beyond these baseline models, demonstrating the effectiveness of its deep learning approach in medical image analysis. In Table 6 a comparative analysis between the previous methodology and the proposed methodology has been given.

**Table 6:** Comparative analysis with the proposed model.

Study	Technique	Accuracy
Pedada et al. (2023)	U-Net Model for Brats 2017 and 2018 dataset segmentation	93.40 and 92.20%
Saeedi et al. (2023)	2D CNN with ensemble machine learning techniques	96.47%
Mahmud et al. (2023)	Redefined CNN Model with modified classification	93.3%
Wang et al. (2022)	Deep CNN on OCT Images	94.90%
Vidyarthi et al. (2022)	CNN with NN Classifier	95.86%
Lamrani et al. (2022)	CNN with Enhanced Classifiers	96%
Yildirim et al. (2023)	Convolutional Neural Network (CNN)-based hybrid model	95.4%
Bacak et al. (2023)	Convolutional Neural Network (CNN) using TensorFlow	90%
Khan et al. (2022)	Deep Learning Models (Convolutional Neural Networks - CNN)	Up to 97.8%
Gómez-Guzmán et al. (2023)	Evaluation of deep convolutional neural network (CNN) models for brain tumor classification	97.12%
Sharma and Shukla (2022)	CNN for brain tumor classification	93.38%
Nayak et al. (2022)	Efficient net on T1 Weighted Data	98.78%
Guan et al. (2021)	Agglomerative Clustering Based Approach	98.04%
Rajput et al. (2024)	VGG19, Inception-v3, and ResNet50	90%
Suryawanshi and Patil (2024)	Convolutional Neural Network (CNN) & VGG19	98.01%
Prasad et al. (2024)	CNN	98.93%
Schiavon et al. (2023)	convolutional neural networks (CNNs)	96%
Rasool et al. (2022)	CNN & SqueezeNet	96.5%
Sarada et al. (2024)	ResNet0V2	96.34%
Rahman and Islam (2023)	Parallel deep convolutional neural networks	98%
Özkaraca et al. (2023)	VGG 16 and basic CNN Architecture	97%
Prabha et al. (2023)	Efficeint Net model Using transfer learning	98.27%
Proposed model	Custom CNN with advance layer arrangement.	99%

The custom architecture and training strategy employed in the proposed model, combined with its remarkable performance metrics, highlight its potential to establish a new standard in the domain of brain tumor classification from MRI scans. The model’s capacity to achieve high accuracy, alongside detailed metrics for different tumor types, offers robust quantitative evidence supporting its adoption and further investigation in clinical environments.

### 4.3 Ablation study

In the ablation study conducted to assess the robustness and significance of each layer within our brain tumor classification model, we systematically eliminated layers and documented the resulting effects on model performance. Initial results indicated a moderate degree of robustness, with the overall accuracy slightly declining from 0.89 after removing one layer to 0.92 after removing up to four layers. The precision, recall, and F1-score for each tumor type demonstrated only minor fluctuations, suggesting that the model preserves its discriminatory power up to a certain depth. However, a stark degradation was observed when all layers were removed, plummeting the overall accuracy to 0.69. This highlights the layers’ collective importance in achieving high diagnostic accuracy. Conversely, the proposed model, which integrates all layers, displayed exceptional performance, achieving near-perfect precision and recall across all categories and culminating in an exemplary overall accuracy of 0.99. The comparison between the layer-ablated versions and the complete model underscores the intricate balance between model depth and performance, as summarized in the following Table 7.

**Table 7:** Ablation study.

		Precision	Recall	F1-Score
**After removing 1 layer**	Glioma	0.98	0.73	0.84
	Meningioma	0.77	0.8	0.79
	No tumor	0.91	0.99	0.95
	Pituitary	0.91	1	0.95
	Overall accuracy = 0.89			
**After removing 2 layers**	Glioma	0.97	0.76	0.85
	Meningioma	0.79	0.87	0.83
	No tumor	0.93	0.99	0.96
	Pituitary	0.96	0.99	0.98
	Overall accuracy = 0.91			
**After removing 3 layers**	Glioma	0.96	0.81	0.88
	Meningioma	0.82	0.87	0.84
	No tumor	0.93	0.99	0.96
	Pituitary	0.97	0.99	0.98
	Overall accuracy = 0.92			
**After removing 4 layers**	Glioma	0.96	0.82	0.89
	Meningioma	0.82	0.85	0.83
	No tumor	0.92	0.99	0.96
	Pituitary	0.97	0.99	0.97
	Overall accuracy = 0.92			
**After removing all layers**	Glioma	0.64	0.53	0.58
	Meningioma	0.52	0.37	0.43
	No tumor	0.79	0.9	0.84
	Pituitary	0.72	0.91	0.8
	Overall accuracy = 0.69			
**Proposed model**	Glioma	1	0.96	0.98
	Meningioma	0.96	0.98	0.97
	No Tumor	1	1	1
	Pituitary	0.99	1	0.99
	Overall accuracy = 0.99			

The ablation study conducted to assess the robustness and significance of each layer within our brain tumor classification model revealed insightful findings. We systematically removed layers and observed the resulting effects on model performance. Interestingly, the model displayed a moderate degree of robustness, with only minor fluctuations in precision, recall, and F1-score when one to four layers were eliminated. However, a significant drop in accuracy was observed when all layers were removed, highlighting the collective importance of the layers in achieving high diagnostic accuracy. Conversely, the proposed model, which integrated all layers, exhibited exceptional performance, with near-perfect precision and recall across all categories and an exemplary overall accuracy of 0.99. This comparison underscores the delicate balance between model depth and performance, emphasizing the critical role of each layer in optimizing classification outcomes.

The comprehensive model clearly demonstrates the necessity of each layer, offering a robust framework for accurate brain tumor classification.

## 5 Discussion

The outcomes yielded by the proposed model are highly encouraging, signifying a notable advancement in leveraging convolutional neural networks for analyzing brain tumor MRI scans. With an accuracy rate of 99%, the model demonstrates exceptional proficiency in distinguishing between various types of brain tumors, as well as accurately identifying non-tumor regions within the brain. Such elevated accuracy holds immense importance in medical diagnostics, where the repercussions of false positives or negatives can be significant.

Moreover, the precision and recall metrics across different tumor types offer a nuanced insight into the model’s performance. The high precision observed for glioma and meningioma indicates that when the model predicts these tumor types, it does so with high reliability. Similarly, the high recall rates indicate the model’s effectiveness in identifying the majority of actual cases for each tumor type, reducing the risk of missed diagnoses.

The F2 score, which emphasizes the importance of recall (minimizing false negatives), is particularly relevant in a medical context (Zhou et al., 2023). A high F2 score, as achieved by proposed model, underscores the model’s capability in correctly identifying positive cases, a critical aspect when early detection can significantly influence treatment outcomes.

Proposed research introduces several innovative elements to the domain of medical imaging, particularly in how deep learning can be tailored to enhance diagnostic precision. The network architecture’s design, which integrates deep convolutional layers with advanced regularization and normalization techniques, is specifically optimized for the complex task of brain tumor identification and classification. This bespoke approach, which diverges from the application of generic CNN models, is a significant contributor to the model’s success.

The impact of these findings extends beyond the technical domain, potentially revolutionizing how brain tumors are diagnosed and classified in clinical settings. By providing a tool that can rapidly and accurately analyze MRI scans, proposed model could assist radiologists in making more informed decisions, facilitating early and accurate diagnoses, and ultimately improving patient care and outcomes.

The model’s performance, while tested on a robust dataset, might still be limited by the diversity and volume of the data available. Real-world applicability will require continual testing and validation on a broader array of MRI scans from diverse patient demographics and equipment.

**Figure 13:**
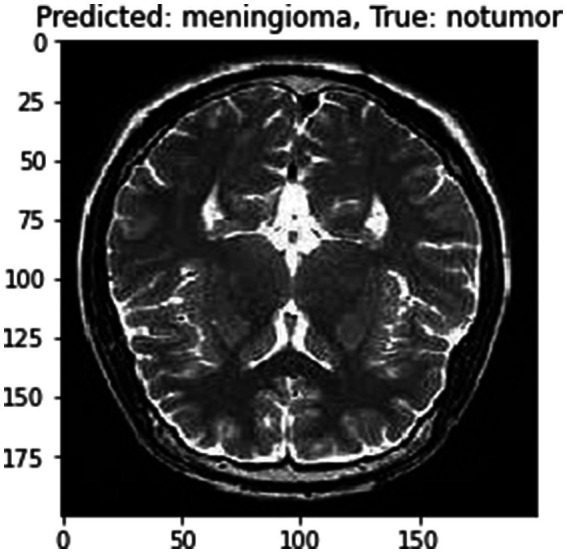
Misclassified instances.

**Figure 14:**
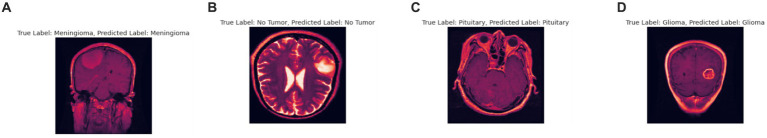
Correct predictions.

Although the model achieves high accuracy, it’s important to acknowledge the potential limitation posed by the “black box” nature of deep neural networks (Salahuddin et al., 2022) Integrating attention mechanisms or employing explainable AI frameworks could significantly enhance the interpretability of the proposed model, thereby increasing its clinical utility. These techniques offer insights into the model’s decision-making process, providing clinicians with a deeper understanding of how and why specific diagnoses are made (Jiang et al., 2023). By elucidating the rationale behind the model’s predictions, these methods can improve trust and confidence in its outputs, ultimately facilitating more informed clinical decision-making. The current version of proposed model is optimized for a specific MRI dataset. Its ability to generalize across different MRI machines and imaging modalities remains to be thoroughly tested. Future work could focus on expanding the model’s adaptability to various imaging conditions, enhancing its robustness and applicability (Chaudhary et al., 2024). The model primarily focuses on cross-sectional MRI data. Incorporating longitudinal and multi-modal imaging data, such as merging MRI with CT or PET scans, has the potential to offer a more holistic understanding of tumor features and development, thereby enriching diagnostic capabilities (Sharma and Chaudhary, 2023). In Figure 13 one instance which was misclassified has been given followed by correct predictions in Figure 14.

The proposed model demonstrates significant advancements in brain tumor MRI analysis, a conscious effort to address these limitations through continuous research and iterative refinement will be essential. Enhancing data diversity, interpretability, and cross-modality generalization will be crucial steps in evolving proposed model from a promising model to a reliable tool in clinical practice.

However, there are major limitations to consider. The model’s performance, while tested on a robust dataset, might be limited by data diversity and volume. Real-world applicability will require validation on a broader array of MRI scans from diverse demographics and equipment. Additionally, the “black box” nature of deep neural networks poses interpretability challenges. Integrating explainable AI techniques could enhance the model’s transparency and clinical utility. Future work should focus on enhancing data diversity, interpretability, and cross-modality generalization, along with extensive clinical validation for integration into clinical workflows. Furthermore, exploring the integration of multimodal imaging data and adapting the model to different populations or tumor types represents promising directions for future research.

One of the significant challenges in enhancing the generalization of datasets for brain tumor classification using MRI scans is the diversity and variability inherent in medical imaging data. MRI scans can vary widely in terms of imaging protocols, machine calibration, and patient demographics, all of which can influence the appearance of the images and, consequently, the performance of classification models. Additionally, the limited availability of labeled medical images due to privacy concerns and the cost of expert annotation poses a challenge for training robust models.

To make the dataset more generalized and comprehensive, it is crucial to include a broader array of MRI scans from diverse populations and multiple healthcare settings. Incorporating images from different MRI machines and including variations in scan settings can help the model learn to recognize tumors across different imaging conditions. Extending the dataset to include multi-modal imaging data, such as combining MRI scans with CT or PET scans, can enrich the dataset and provide more comprehensive features for the model to learn. This approach can improve diagnostic accuracy and help the model generalize better to new, unseen cases. Furthermore, synthetic data generation techniques like Generative Adversarial Networks (GANs) can be employed to augment the dataset, providing a wider array of training examples without compromising patient privacy. These strategies would enhance the model’s robustness and its applicability in diverse clinical environments.

The integration of our CNN model into clinical workflows could significantly enhance the diagnostic process by providing rapid, preliminary analysis of MRI scans. This tool could serve as a second opinion to assist radiologists in identifying subtle or ambiguous tumor signs, potentially speeding up the diagnosis and reducing the likelihood of human error (Chaudhary and Agrawal, 2021). Challenges for integration include the need for extensive clinical validation to ensure accuracy and reliability, as well as adjustments to existing medical software systems to accommodate the new AI capabilities.

Future research could explore the integration of multimodal imaging data, combining MRI with CT or PET scans to enrich the dataset and potentially improve diagnostic accuracy. Additionally, further studies could focus on adapting the model to different populations or other types of tumors, enhancing its applicability. Another promising direction is the incorporation of explainable AI techniques to provide insights into the model’s decision-making processes, increasing its transparency and trustworthiness for clinical use.

## 6 Conclusion

This study advances the application of CNNs in the classification of brain tumors from MRI scans, demonstrating a significant improvement over existing methods. The customized CNN architecture introduced novel aspects tailored specifically for medical imaging, setting a new benchmark for accuracy and efficiency in this field. Tailored specifically for the nuanced task of brain tumor classification, proposed method demonstrated an impressive 99% accuracy rate in proposed study, alongside high precision and recall across various tumor categories, underscoring its potential as a robust diagnostic aid in clinical settings. The implications of these discoveries are significant for the realm of medical imaging and diagnostics. The capability of the model to precisely classify brain tumors from MRI scans has the potential to transform diagnostic procedures, leading to heightened accuracy, shortened analysis durations, and potentially better patient outcomes by enabling earlier and more accurate diagnoses. This research emphasizes the value of developing specialized, task-specific AI models for medical imaging, which can address the unique challenges of the field more effectively than general-purpose models.

Looking ahead, there are several promising directions for future research. Expanding the diversity of the training and validation datasets can enhance model’s generalizability and robustness. Improving the model’s interpretability would make it more valuable in clinical contexts, where understanding the basis for its predictions is crucial. Extending its capabilities to multi-modal and longitudinal analyses could offer deeper insights into tumor progression and response to treatment. Finally, rigorous clinical validation and integration into clinical workflows will be essential steps toward realizing proposed model’s potential to improve diagnostic practices and patient care in the realm of brain tumor treatment. By pursuing these avenues, we can build on the solid foundation laid by this study to further advance the application of AI in medical diagnostics, ultimately contributing to better health outcomes and enhanced clinical decision-making.

## Data availability statement

The original contributions presented in the study are included in the article/supplementary material, further inquiries can be directed to the corresponding author.

## Author contributions

EA: Conceptualization, Investigation, Writing – original draft. AT: Data curation, Methodology, Software, Writing – original draft. DD: Formal analysis, Supervision, Writing – review & editing. SB: Conceptualization, Writing – original draft. TM: Resources, Visualization, Writing – review & editing. AA: Funding acquisition, Validation, Writing – review & editing. KA: Formal analysis, Project administration, Writing – review & editing. MA: Formal analysis, Supervision, Writing – review & editing.
